# TBC1D3, a Hominoid-Specific Gene, Delays IRS-1 Degradation and Promotes Insulin Signaling by Modulating p70 S6 Kinase Activity

**DOI:** 10.1371/journal.pone.0031225

**Published:** 2012-02-13

**Authors:** Marisa J. Wainszelbaum, Jialu Liu, Chen Kong, Priya Srikanth, Dmitri Samovski, Xiong Su, Philip D. Stahl

**Affiliations:** 1 Department of Cell Biology and Physiology, Center for Human Nutrition, Washington University School of Medicine, St. Louis, Missouri, United States of America; 2 Department of Internal Medicine, Center for Human Nutrition, Washington University School of Medicine, St. Louis, Missouri, United States of America; University of Florida, United States of America

## Abstract

Insulin/IGF-1 signaling plays a pivotal role in the regulation of cellular homeostasis through its control of glucose metabolism as well as due to its effects on cell proliferation. Aberrant regulation of insulin signaling has been repeatedly implicated in uncontrolled cell growth and malignant transformations. TBC1D3 is a hominoid specific gene previously identified as an oncogene in breast and prostate cancers. Our efforts to identify the molecular mechanisms of TBC1D3-induced oncogenesis revealed the role of TBC1D3 in insulin/IGF-1 signaling pathway. We document here that TBC1D3 intensifies insulin/IGF-1-induced signal transduction through intricate, yet elegant fine-tuning of signaling mechanisms. We show that TBC1D3 expression substantially delayed ubiquitination and degradation of insulin receptor substrate-1 (IRS-1). This effect is achieved through suppression of serine phosphorylation at S636/639, S307 and S312 of IRS-1, which are key phosphorylation sites required for IRS-1 degradation. Furthermore, we report that the effect of TBC1D3 on IRS-1:S636/639 phosphorylation is mediated through TBC1D3-induced activation of protein phosphatase 2A (PP2A), followed by suppression of T389 phosphorylation on p70 S6 kinase (S6K). TBC1D3 specifically interacts with PP2A regulatory subunit B56γ, indicating that TBC1D3 and PP2A B56γ operate jointly to promote S6K:T389 dephosphorylation. These findings suggest that TBC1D3 plays an unanticipated and potentially unique role in the fine-tuning of insulin/IGF-1 signaling, while providing novel insights into the regulation of tumorigenesis by a hominoid-specific protein.

## Introduction

Knowledge of genes that are specific to humans is likely to shed light on our understanding of human physiology and pathology. TBC1D3 is a hominoid-specific gene that maps to human chromosome 17 [1]. Like many hominoid-specific genes, TBC1D3 appears to have evolved in the primate lineage by segmental duplication [2]. Recent work indicates that TBC1D3 is a multi-copied gene in humans [3], [4], with variable copy numbers in different individuals [5]. Although TBC1D3 is one of the first hominoid-specific genes which have been examined at the protein level [6], [7], little is known about its function. Initial studies indicate that TBC1D3 is linked to cell proliferation and signaling by growth factor receptors. Indeed, signals generated by the EGF receptor (EGFR) are substantially amplified by TBC1D3 expression, in part, because the degradation of the receptor is delayed due to suppressed EGFR ubiquitination. TBC1D3 expression also enhances Ras activation in growth factor-stimulated and unstimulated cells [6].

In this study, we focused on the role of TBC1D3 in regulating the mTOR-S6K-IRS-1 axis within the insulin signaling pathway. Because of its central role in cell proliferation and glucose homeostasis, the signaling pathway controlled by insulin and other growth factors is one of the most intensely investigated cellular pathways. Insulin resistance precedes the development of type 2 diabetes, and in addition, hyper-activation of the pathway is associated with several proliferative diseases [8], [9].

Insulin receptor signaling is mediated by the recruitment of numerous adapters and among the most important is IRS-1. IRS-1-mediated insulin signaling is attenuated by IRS-1 ubiquitination and degradation [10]. Key residues in IRS-1, which are essential for its ubiquitination, include S636/639, a site that is phosphorylated, directly or indirectly, by S6K. S6K, in turn, is phosphorylated and activated by mammalian target of rapamycin (mTOR) [11].

In mammalian cells, at least two proteins are regulated by the activity of mTOR: 4E-BP1 and S6K, which are involved in the initiation of protein translation and are regulated by Ser/Thr phosphorylation [8], [12]. S6K is also regulated by PP2A, a family of abundantly expressed serine-threonine phosphatases that plays an important role in diverse cellular functions [13], [14].

Using insulin-sensitive human cell lines, we report that TBC1D3 expression suppresses IRS-1 ubiquitination and delays degradation by selectively enhancing the dephosphorylation of S6K at T389. We provide evidence that a specific PP2A regulatory subunit B56γ, recently shown to dephosphorylate S6K at T389 [15], is a target of TBC1D3. TBC1D3 delays IRS-1 degradation by accelerating the dephosphorylation/inactivation of S6K thereby enhancing insulin signaling.

This study was aimed at exploring the effect of TBC1D3 on insulin/IGF-1 signal transduction pathway. Our findings indicate that TBC1D3 expression enhances the intensity and duration of the insulin/IGF-1 signaling cascade.

## Results

### Insulin signaling pathway is positively regulated by TBC1D3

**Figure 1:**
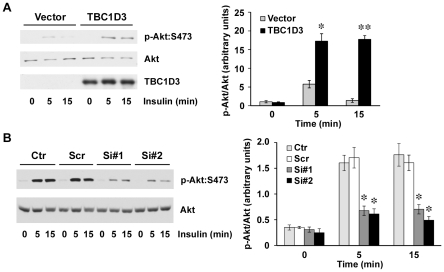
TBC1D3 increases signaling through the activation of insulin pathway. (A) HepG2 cells transfected with myc-TBC1D3 or empty vector were serum-starved, and stimulated with insulin (10 nM) for the indicated times. Phosphorylation and protein levels of Akt were analyzed by Western blotting. (*Right panel*) Quantification data of Akt:S473 phosphorylation normalized to Akt protein levels (* *p*<0.05, ** *p*<0.01). (B) HepG2 cells were transfected with two different TBC1D3 siRNA oligos (50 nM) (si#1 and si#2), scramble siRNA (scr) or untransfected (ctr). After 36 h, cells were serum-starved and stimulated with 10 nM insulin for the indicated times and analyzed by Western blotting with the listed antibodies. (*Right panel*) Quantification data of Akt:S473 phosphorylation normalized to Akt protein levels (* *p*<0.05). The data are presented as means ± SD of three independent experiments.

In previous studies, we demonstrated that TBC1D3 expression has a powerful effect on cell proliferation that is further enhanced by epidermal growth factor (EGF) [6]. To determine whether TBC1D3 modulates signal transduction through the insulin receptor, we examined insulin signaling in HepG2 cells (American Type Culture Collection, Manassas, VA), a well-studied human hepatocellular carcinoma cell line. HepG2 cells were transfected with myc-TBC1D3 and incubated with 10 nM insulin. Phosphorylation levels of Akt on S473, a well-established downstream marker for activation of insulin receptor signaling, were monitored by Western blotting. Total cellular contents of Akt were used to normalize the results in each sample. Figure 1A shows that insulin-induced phosphorylation of Akt:S473 is significantly increased in cells expressing TBC1D3, suggesting increased activation of insulin signaling.

To exclude misleading phenotypes due to over-expression, we utilized RNA interference to suppress endogenous TBC1D3. Two different siRNAs directed against TBC1D3 (siRNA#1 and siRNA#2) were transfected into HepG2 cells. A scrambled, irrelevant siRNA (scr) was used as a negative control. 36 hours later, cells were serum-starved and stimulated with insulin (10 nM).

We were not able to detect the expression of endogenous TBC1D3 protein, since a reliable anti-TBC1D3 antibody is currently unavailable. Thus, a quantitative real-time PCR was carried out to determine the efficiency of siRNA-mediated target suppression. Both siRNA duplexes decreased mRNA levels of TBC1D3 by 60%–70% (data not shown). No off-target silencing effect was observed.

Consistent with the results obtained when over-expressing TBC1D3, silencing of TBC1D3 abrogated the activation of Akt following insulin stimulation (Figure 1B). Collectively, these results establish that TBC1D3 is a positive regulator of the insulin signaling pathway.

### TBC1D3 modulates IRS-1 phosphorylation

The insulin receptor (IR) is activated by autophosphorylation on tyrosine residues that function as docking sites for adaptor proteins. Among these are the insulin receptor substrate (IRS) proteins. IRS-1, which is widely expressed, is the most-well-characterized member of the IRS family. IRS proteins are rapidly recruited to the insulin receptor and activated by tyrosine phosphorylation following insulin stimulation [16], [17]. While phosphorylation on tyrosine residues is required for IRS-1 activation, serine phosphorylation plays an important role in both the propagation and the attenuation of insulin signaling [18]–[20].

To gauge the effect of TBC1D3 expression on IRS-1 phosphorylation, IRS-1 and myc-TBC1D3 were co-expressed in Hek293 cells (American Type Culture Collection, Manassas, VA). Total cell lysates were prepared after insulin treatment and IRS-1 phosphorylation status was analyzed by Western blotting using phospho-specific antibodies. Total IRS-1 levels were measured and used to normalize and quantify the results in each sample.

**Figure 2:**
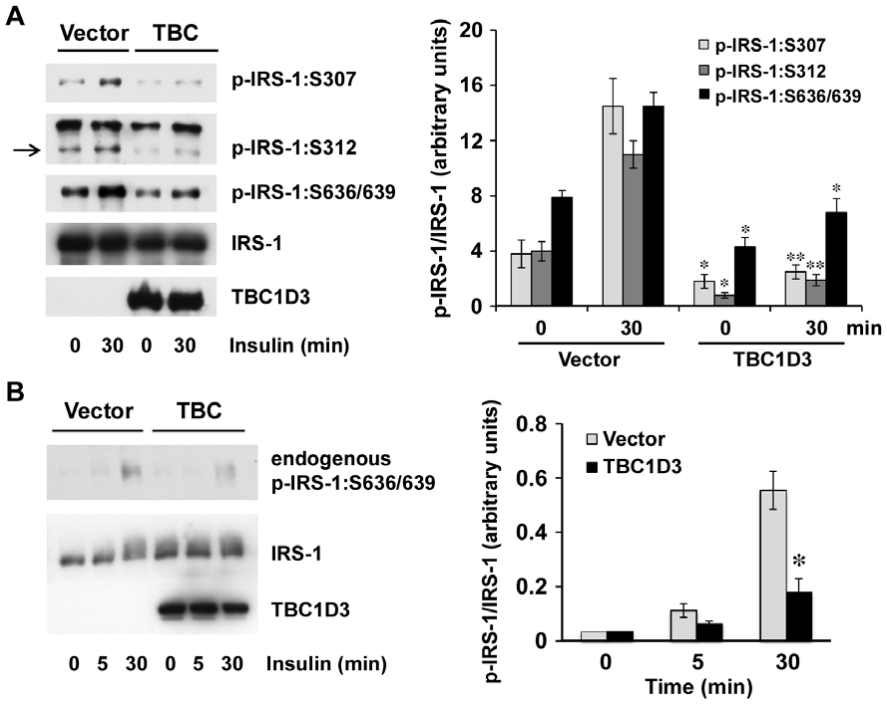
TBC1D3 selectively decreases IRS-1 Serine phosphorylation. (A) Empty vector or myc-TBC1D3 was co-transfected with pCIS2-IRS-1 in Hek293 cells. Cells were stimulated with insulin (10 nM) for 30 min. Phosphorylation and protein levels of IRS-1 were analyzed by Western blotting. (*Right panel*) Quantification data of IRS-1 phosphorylation normalized to IRS-1 protein levels (* *p*<0.05, ** *p*<0.01). (B) Hek293 cells were transfected with myc-TBC1D3 or empty vector, serum-starved and stimulated with insulin (10 nM) for 5 or 30 min. Phosphorylation of endogenous IRS-1:S636/639 was analyzed by Western blotting. (*Right panel*) Quantification data of endogenous IRS-1 phosphorylation normalized to IRS-1 protein levels (* *p*<0.05). The data are presented as means ± SD of three independent experiments.

Over-expression of TBC1D3 did not affect insulin-stimulated IRS-1 phosphorylation on tyrosine residues (data not shown), however it led to a reduced phosphorylation of recombinant IRS-1 on S307, S312 and S636/639 in response to insulin, when compared to control cells (Figure 2A). These observations were confirmed by measurements of endogenous IRS-1: S636/639 phosphorylation in TBC1D3-expressing cells, which was significantly lower after 5 or 30 min of insulin stimulation (Figure 2B). Similar data were obtained on endogenous IRS-1 phosphorylation when cells were treated with IGF-1 instead of insulin (data not shown). However, TBC1D3 appeared to have no effect on IRS-1: S1101 phosphorylation, showing a phosphorylation pattern similar to control cells (data not shown).

A recent publication by Ye and colleagues, found IRS-1:S270 as a novel IRS-1 phosphorylation site that may be involved in regulating the phosphorylation of other serine residues by TNF-α [21]. Our results demonstrate that insulin stimulation of phosphorylation at IRS-1:S270 is also significantly reduced in TBC1D3-over-expressing cells. Collectively, these findings indicate that IRS-1 serine phosphorylation, but not tyrosine phosphorylation, is selectively reduced in the presence of TBC1D3.

### TBC1D3 over-expression delays IRS-1 ubiquitination and degradation

To understand the functional consequences of decreased IRS-1 serine phosphorylation by TBC1D3 over-expression, we next studied the kinetics of IRS-1 degradation. To test whether TBC1D3 delays this process, DU145 cells (American Type Culture Collection, Manassas, VA) transiently transfected with myc-TBC1D3 or empty vector were serum-starved, and incubated with insulin for the indicated times. Total cellular levels were quantified by Western blotting and were used to normalize the amount of protein remaining at each time point. Cycloheximide (25 µg/ml) was added to block *de novo* IRS-1 synthesis.

**Figure 3:**
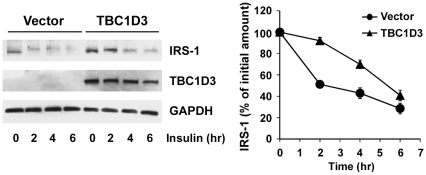
TBC1D3 expression blocks IRS-1 degradation. IRS-1 degradation is delayed in cells expressing TBC1D3. DU145 cells transfected with myc-TBC1D3 or empty vector were serum-starved, and stimulated with insulin (10 nM) for the indicated times. Protein levels of IRS-1 were analyzed by Western blotting. (*Right panel*) Quantification data of IRS-1 normalized to GAPDH protein levels. The value of IRS-1 at time 0 was set at 1.0. The data are presented as means ± SD of three independent experiments.

Control cells showed a rapid degradation of IRS-1; about 50% of the IRS-1 signal was lost after 2 hours of insulin treatment (Figure 3). However, IRS-1 degradation was significantly delayed in cells expressing TBC1D3; only about 10% of the IRS-1 protein was degraded after 2 hours, and only after 4 hours was there a substantial reduction comparable to control cells (∼50%).

In agreement with these results, over-expression of TBC1D3 has also resulted in suppression of IRS-1 ubiquitination (data not shown), suggesting that TBC1D3 regulates IRS-1 turnover via modulation of its ubiquitination status.

### p70 S6K activation is reduced in TBC1D3 expressing cells

**Figure 4:**
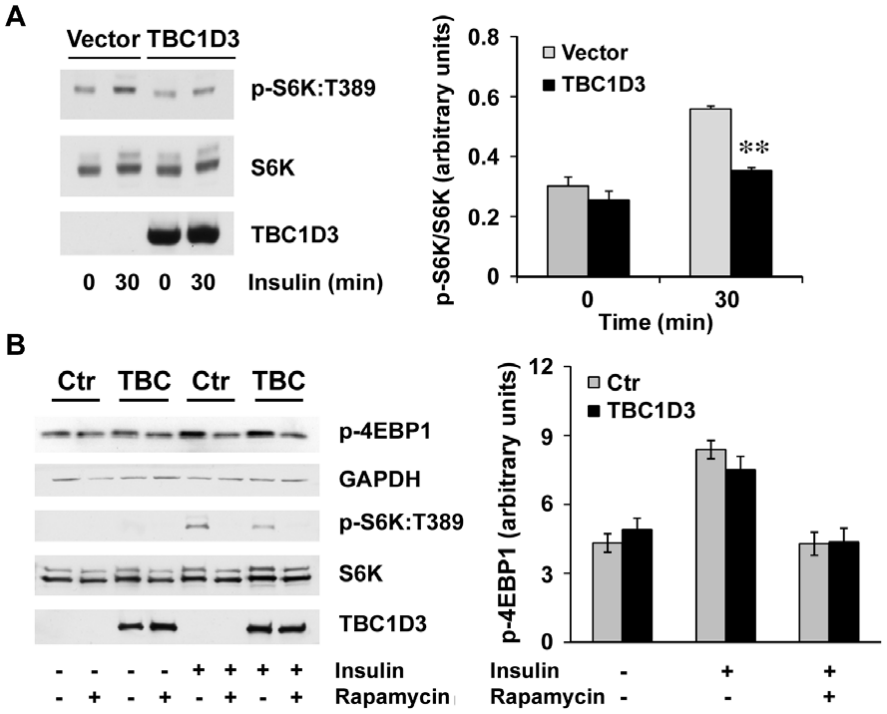
TBC1D3 reduces S6K activation, but does not affect mTOR-C1 pathway. (A) HepG2 cells transfected with myc-TBC1D3 or empty vector were serum-starved, and stimulated with insulin (10 nM) for 30 min. Phosphorylation and protein levels of S6K were analyzed by Western blotting. (*Right panel*) Quantification data of S6K:T389 phosphorylation normalized to S6K protein levels (** *p*<0.01). (B) HepG2 cells transfected with myc-TBC1D3 or empty vector were serum-starved, pre-treated with Rapamycin (50 nM) for 2 h, and stimulated with insulin (10 nM) for 30 min. Phosphorylation and protein levels of 4EBP1 and S6K were analyzed by Western blotting. (*Right panel*) Quantification data of 4EBP1 phosphorylation normalized to GAPDH protein levels. The data are presented as means ± SD of three independent experiments.

mTOR is a serine/threonine protein kinase that regulates cell growth and proliferation through a rapamycin-sensitive pathway involving the regulatory proteins p70 S6K and the eukaryotic initiation factor 4E-binding protein-1 (4EBP1) [11]. Several independent studies reported that S6K is involved in the phosphorylation of IRS-1 at multiple serine residues, including S307, S312, S270 and S636/639 [21]–[24]. To explore the mechanism underlying the decreased IRS-1 phosphorylation in TBC1D3-expressing cells, we first examined the ability of insulin to stimulate phosphorylation of S6K at T389, a residue that is critical for its activation [25]. As shown in Figure 4A, insulin stimulation of S6K:T389 phosphorylation is robust, increasing almost two-fold after 30 min of stimulation. However, in TBC1D3-expressing cells, S6K:T389 phosphorylation in response to insulin is reduced. The effect of TBC1D3 on S6K:T389 phosphorylation is modest, however it is statistically significant. These findings suggested that the mTOR signaling pathway may be involved in the reduced serine phosphorylation and degradation of IRS-1, through a defect in S6K activation.

To support this hypothesis, we next determined whether mTOR, the kinase responsible for S6K activation, was defective by evaluating the phosphorylation status of the other mTOR substrate, 4EBP1. However, phosphorylation of 4EBP1 was normal and similar in both TBC1D3-expressing and control cells after insulin treatment (Figure 4B). Moreover, when the cells were pre-treated with rapamycin for 2 h before insulin stimulation, both S6K and 4EBP1 phosphorylation signals were completely quenched, confirming that mTOR pathway was not defective and fully functional under these experimental conditions.

### TBC1D3 regulates PP2A activity

**Figure 5:**
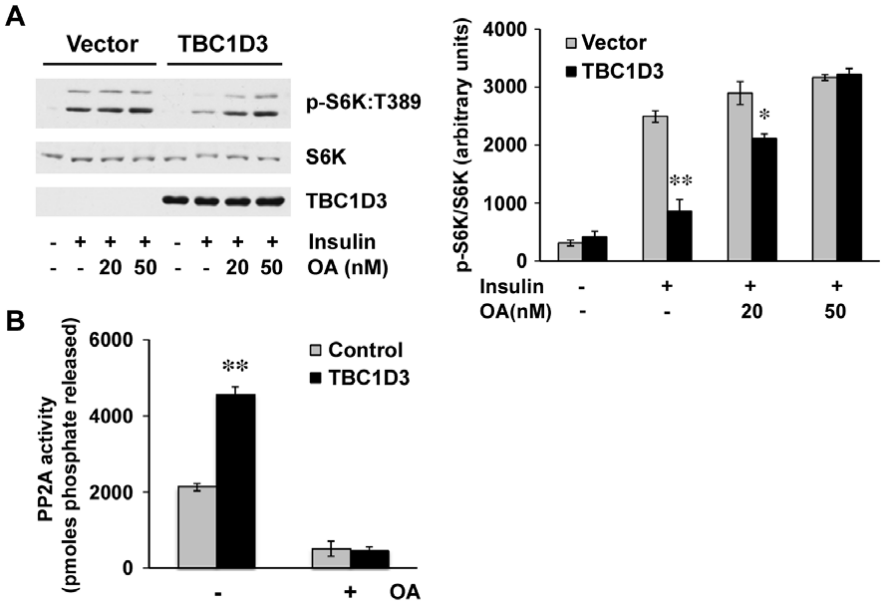
PP2A activity is increased in TBC1D3-expressing cells. (A) HepG2 cells transfected with myc-TBC1D3 or empty vector were serum-starved, and pre-treated with OA at various concentrations for 30 min, prior to insulin stimulation. Phosphorylation and protein levels of S6K were analyzed by Western blotting. (*Right panel*) Quantification data of S6K:T389 phosphorylation normalized to S6K protein levels (* *p*<0.05, ** *p*<0.01). (B) HepG2 cells transfected with myc-TBC1D3 or empty vector were serum-starved, and pre-treated with or without OA (50 nM) for 30 min before insulin stimulation. PP2A activation was assayed *in vitro* using the synthetic phosphopeptide K-R-pT-I-R-R, as described in Experimental Procedures (** *p*<0.01). The data are presented as means ± SD of three independent experiments.

Reduced S6K phosphorylation in cells expressing TBC1D3 could be due to decreased mTOR kinase activity or increased phosphatase activity. Several recent studies have linked PP2A to the regulation of S6K [13], [15], [26], [27]. To determine if TBC1D3 modulates PP2A activity on S6K, HepG2 cells were transfected with myc-TBC1D3 and pre-treated with okadaic acid (OA) before insulin stimulation. OA is a cell permeable inhibitor that selectively inhibits PP2A when used at low concentrations (IC_50_ = 0.51 nM) [28], [29]. The analysis of phospho-S6K revealed that, as in previous results, S6K activation was significantly reduced in TBC1D3-expressing cells, when compared to control cells (Figure 5A). Treatment with OA dramatically increased phosphorylation, restoring it to the control levels (Figure 5A, bar graph). The concentrations assayed (20 and 50 nM) were too low to have any effect over PP1, another phosphatase of the same family [28]. Additionally, we were able to exclude the involvement of PP2B/calcineurin by using specific inhibitors FK506 and Calyculin A (data not shown).

Direct verification of PP2A enhanced activation was detected *in vitro* using a specific synthetic phosphopeptide (Figure 5B). PP2A enzyme immunoprecipitated from TBC1D3-expressing cells showed a much higher phosphatase activity, relative to that in control cells (>2.5-fold increase). The activity was almost completely abolished when the cells were pre-treated with OA, confirming that the PP2A activity was specifically modulated by TBC1D3 expression. Biochemical experiments were used to rule out enhanced expression levels of the different PP2A subunits when over-expressing TBC1D3 (data not shown).

### TBC1D3 modulates the effect of PP2A B56γ on S6K:T389 phosphorylation

**Figure 6:**
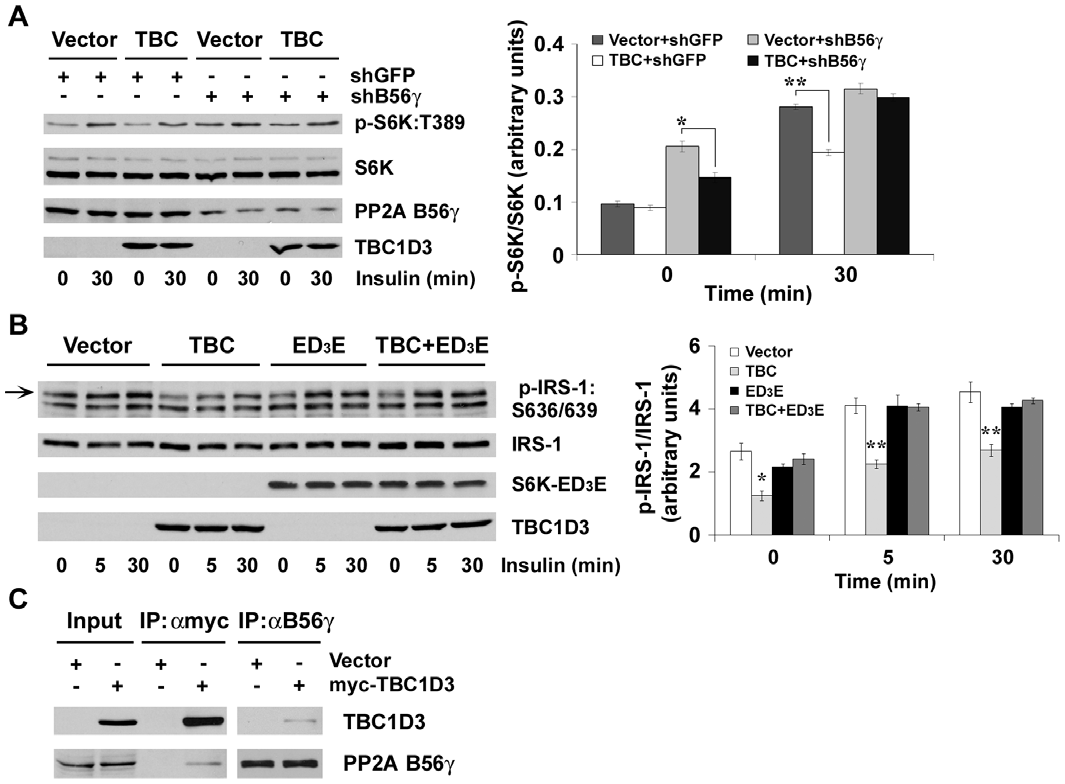
PP2A B56γ mediates S6K phosphorylation in response to TBC1D3 expression. (A) DU145 cells with GFP (control) or PP2A B56γ knockdown were transfected with myc-TBC1D3 or empty vector, serum-starved, and stimulated with insulin (10 nM) for 30 min. Phosphorylation and protein levels of S6K were analyzed by Western blotting. (*Right panel*) Quantification data of S6K:T389 phosphorylation normalized to S6K protein levels (* *p*<0.05, ** *p*<0.01). (B) Reduction of IRS-1:S636/639 phosphorylation induced by TBC1D3 expression is rescued by a rapamycin-resistant mutant of S6K. pCIS2-IRS-1, myc-TBC1D3 or empty vector were co-transfected with HA-S6K-ED_3_E or vector in DU145 cells. Cells were stimulated with insulin (10 nM) for 0, 5 or 30 min after serum-starvation. Phosphorylation and protein levels of IRS-1 were analyzed by Western blotting. (*Right panel*) Quantification data of IRS-1:S636/639 phosphorylation normalized to IRS-1 protein levels (* *p*<0.05, ** *p*<0.01). The data are presented as means ± SD of three independent experiments. (C) TBC1D3 co-immunoprecipitates with PP2A B56γ. Hek293 cells were transfected with myc-TBC1D3 or empty vector. Cell lysates were immunoprecipitated with anti-myc or anti-B56γ antibodies, respectively. Co-IP samples were separated by SDS-PAGE and analyzed by Western blotting with specific antibodies.

PP2A B56γ, a regulatory B′ subunit of PP2A encoded by *PPP2R5C* gene, has been identified as a specific negative regulator of S6K phosphorylation [15]. To explore whether TBC1D3 modulates PP2A activity on S6K phosphorylation through the specific PP2A regulatory subunit B56γ, PP2A B56γ-knockdown DU145 cells were prepared using specific shRNAs. Western blotting results indicated that PP2A B56γ protein expression was suppressed by nearly 70% in the presence of three independent PP2A B56γ shRNAs compared with control shRNAs (Figure 6A). No off-target silencing effects were observed. Control and PP2A B56γ-silenced cells were transfected with myc-TBC1D3 before insulin stimulation.

In control cells, analysis of S6K:T389 phosphorylation levels indicates that S6K activity is dramatically elevated by insulin treatment as expected, and that S6K:T389 phosphorylation is modestly reduced in the presence of TBC1D3, confirming the results in Figure 4. Consistent with previously published findings [15], a significant increase in the level of phosphorylated S6K protein was observed in the PP2A B56γ-knockdown cells.

Similar to insulin-treated control cells, TBC1D3 expression significantly reduced S6K:T389 phosphorylation in PP2A B56γ-knockdown cells. In the insulin-treated PP2A B56γ-knockdown cells, there was a significant increase in S6K:T389 phosphorylation (compared with the absence of insulin treatment) but TBC1D3 expression was less effective in reducing S6K:T389 phosphorylation.

To confirm the role of S6K in TBC1D3-suppression of IRS-1 phosphorylation, we took advantage of a rapamycin-resistant mutant of S6K, S6K-ED_3_E. We examined the effect of TBC1D3 on IRS-1:S636/639 phosphorylation in cells transfected with IRS-1 and with either TBC1D3 alone or TBC1D3 and S6K-ED_3_E. Following stimulation with insulin, IRS-1 was immunoprecipitated, resolved by SDS-PAGE and blotted with the specific antibody against phospho-IRS-1:S636/639. The results are presented in Figure 6B. TBC1D3 suppressed the phosphorylation of IRS-1 at S636/639 before and after insulin treatment. However, following transient expression of the S6K-ED_3_E construct, expression of TBC1D3 was unable to suppress insulin-stimulated IRS-1:S636/639 phosphorylation indicating that S6K is the likely target of TBC1D3.

We speculated that TBC1D3 modulates the effect of PP2A B56γ on S6K:T389 phosphorylation by functionally interacting, directly or indirectly, with PP2A B56γ. To verify this possibility, a reciprocal co-immunoprecipitation experiment was carried out to determine whether TBC1D3 interacts with endogenous B56γ. Hek293 cells transfected with empty vector or myc-TBC1D3 were lysed and immunoprecipitated with anti-myc or anti-B56γ antibodies followed by SDS-PAGE and Western blotting. As shown in Figure 6C, endogenous B56γ was specifically pulled down in TBC1D3-expressing samples incubated with anti-myc antibody, but not control Hek293 cells, while TBC1D3 was precipitated in TBC1D3-expressing samples incubated with anti-B56γ antibody. These results suggest that TBC1D3 and B56γ interact with each other while pointing to the need for further studies to define the structural basis for functional interactions.

## Discussion

TBC1D3 appears to have evolved along the hominoid-lineage 35 million years ago by segmental duplication [2]. It was identified by a set of innovative experiments involving human DNA transfected into and carried by mouse tumors [30] and shown subsequently to be an oncogene by Pei *et al* [1]. More recently, Hatanaka *et al* [31] using a human gall bladder cancer cDNA library, identified TBC1D3 as an oncogene. Hodzic *et al* [3] and Paulding *et al* [2] identified multiple transcripts of TBC1D3 in humans and revealed that multiple TBC1D3 paralogs are expressed in insulin-sensitive, as well as other tissues. Recent work by Eichler and colleagues [5] on copy number variation in a large cohort of humans from different ethnic backgrounds revealed a surprising finding – European genomes encode ∼8–20 copies of TBC1D3, Asian genomes ∼15–30 copies and African (Yoruba) genomes ∼30–55 copies. The importance of this is unclear but it seems reasonable to speculate that TBC1D3 has been subjected to some unknown positive evolutionary pressure. Although absent from the mouse and other model organisms, TBC1D3 is found in the Chimp genome as a single copy gene [4]. Interestingly, the Neanderthal draft genome encodes TBC1D3 [32] although apparently also at low copy number. Our initial work [6] and that of Fritolli *et al* [7], indicates that TBC1D3 expression enhances cell proliferation and alters the signaling properties of growth factor receptors. EGF-stimulated pinocytosis was increased [7] and EGFR trafficking and degradation were delayed [6] suggesting that phosphorylation and/or ubiquitination of the EGFR were altered by TBC1D3 expression.

There has been a growing realization that signaling abnormalities within nutrient signaling pathways can lead to abnormal cell growth and cancer. The insulin/IGF-1 signaling pathway and the mTOR/S6K cascade defines a metabolic network that integrates glucose metabolism and energy balance with cell proliferation and survival [33]. Our current study provides evidence for a regulatory role for TBC1D3 in insulin signaling with a putative link to cell proliferation.

We report that TBC1D3 expression enhances the signaling properties of insulin receptor as determined by standard assay, the phosphorylation of Akt. Importantly, silencing of TBC1D3 suppressed the activation of Akt by insulin. Degradation of endogenous IRS-1 was suppressed following insulin treatment in cells expressing TBC1D3 (Figure 3). Since serine phosphorylation is key to IRS-1 degradation, these experiments led us to consider IRS-1 phosphorylation as a prime TBC1D3 target. More than a dozen different kinases are known to phosphorylate IRS-1 in addition to receptor kinase-driven tyrosine phosphorylation [34]. These phosphorylation sites orchestrate IRS-1 interaction with growth factor receptors, its activation of Ras via binding of Grb2 and SOS [35], [36] and its translocation to the nucleus [37].

IRS-1 degradation following ubiquitination is part of the negative feedback loop designed to initiate and limit nutrient uptake and growth [20]. S6K phosphorylates IRS-1 on S636/639, a key residue for IRS-1 degradation. It remains unclear whether IRS-1:S636/639 is phosphorylated directly by S6K or not. However, phosphorylation at IRS-1:S270 has been reported to favor subsequent phosphorylation on S307, S636/639, and S1101 of IRS-1 in response to insulin stimulation (although S1101 may be phosphorylated by PKC θ). Using site-specific antibodies, we found that TBC1D3 expression suppressed serine phosphorylation at all of the above sites except for IRS-1:S1101.

In their pioneering study, Pan and colleagues [10] reported that Cul7 ubiquitinates IRS-1 and that phosphorylation of IRS-1 by mTOR/S6K is important for Cul7 recognition of IRS-1. These findings have placed IRS-1 and Cul7 in the center of a major growth factor signaling and cell proliferation pathway [16], [34], [38], [39]. The physiological importance of IRS-1 goes well beyond that of insulin and IGF signaling. It is associated more broadly with growth and shown to facilitate tumorigenesis. IRS-1 also operates as a signal-transducing element in the nucleus where it binds to upstream binding factor 1, which is involved in rRNA synthesis [37]. Over-expression of IRS-1 has been associated with breast cancer, and estrogen has been shown to enhance transcription/translation of IRS-1 [40]–[42].

To explore the mechanisms of TBC1D3-induced suppression of IRS-1 phosphorylation, we focused on the effect of TBC1D3 on S6K activation. S6K is activated by mTOR signaling via phosphorylation on T389 and inactivated by PP2A-dependent dephosphorylation. Expression of TBC1D3 can suppress phosphorylation and/or enhance dephosphorylation of S6K at T389.

To test the hypothesis that TBC1D3 enhances the dephosphorylation and inactivation of S6K in PP2A-dependent manner, we took advantage of the potent PP2A inhibitor OA. OA specifically inhibits the protein phosphatases 1 (PP1) and 2A (PP2A) [28]. PP2A is inhibited by OA at concentrations of 1–2 nM, whereas PP1 is much less sensitive. OA is also cell-permeable permitting its use in intact cells [1], [3]. As shown in Figure 5A, OA reversed the inhibitory effect of TBC1D3 on S6K dephosphorylation at T389, suggesting that PP2A is a target of TBC1D3.

PP2A is a large family of heterotrimeric enzymes that account for the majority of total serine/threonine phosphatase activity in most tissues and cells [43]. Although a dimer comprised of a 65-kDa scaffolding (A) subunit and a 36-kDa catalytic (C) subunit constitutes the core enzymatic activity of PP2A, the binding of a third regulatory (B) subunit to the AC core enzyme regulates PP2A activity, cellular localization, and substrate specificity [43]. PP2A can exist in cells as either the AC core complex or an ABC heterotrimeric holoenzyme that is the dominant form in most cells. In humans there are at least 16 B regulatory subunits that can be classified into B (B55), B′ (B56), B″, and B‴ families based on sequence similarity [44]. Studies by Chen *et al* [45] showed that down-regulation of B56γ together with expression of the SV40 small T antigen (ST) reduced PP2A activity by 43%, suggesting PP2A-B56γ holoenzyme may represent a substantial proportion of the total PP2A activity *in vivo*. The assembly and function of the active PP2A complex is regulated by methylation [46], by phosphorylation and by factors such as the protein a4 [47] and ST [44]. It is possible that TBC1D3 alters any one of these regulatory post-translational modifications by displacing one or more factors known to interact with PP2A to enhance its effect on PP2A-dependent dephosphorylation of S6K:T389.

A provocative study by Hahn *et al* [15] showed that *PPP2R5C*, encoding the B56γ regulatory subunit of PP2A, acts as a specific negative regulator of S6K phophorylation in flies and humans. This discovery prompted us to examine to role of PP2A B56γ in TBC1D3 suppression of S6K activation. As shown in Figure 6A, silencing of B56γ resulted in enhanced S6K:T389 phosphorylation (confirming earlier reports). TBC1D3 expression significantly reduced S6K:T389 phosphorylation in these cells. S6K:T389 phosphorylation in PP2A B56γ-silenced cells was further enhanced by insulin treatment. However, TBC1D3 was much less effective in reducing S6K:T389 phosphorylation in PP2A B56γ-silenced cells. These findings suggest that insulin was more effective in driving S6K:T389 phosphorylation than TBC1D3 was in enhancing dephosphorylation at low levels of PP2A B56γ.

To confirm that the target of TBC1D3 was S6K and not a downstream event (e.g., the direct dephosphorylation of IRS-1:S636/639 by PP2A), we took advantage of a rapamycin-resistant mutant of S6K called S6K-ED_3_E [48]. Expression of S6K-ED_3_E permitted the insulin sensitive stimulation of IRS-1:S636/639 phosphorylation but abolished the TBC1D3 inhibitory effect. Clearly, other unknown factors are at play such as the effect of TBC1D3 expression on overall PP2A activity (Figure 5B). Human PP2A B56γ has at least three differentially spliced isoforms, B56γ1, B56γ2, and B56γ3 [49] and even though the introduction of specific shRNAs should target all three isoforms, we were unable to confirm the knockdown of B56γ1 since the antibody used in our experiments for detecting B56γ only recognizes the B56γ2 and B56γ3 isoforms. However, we confirmed the interaction between TBC1D3 and B56γ by co-immunoprecipitation assays (Figure 6C).

**Figure 7:**
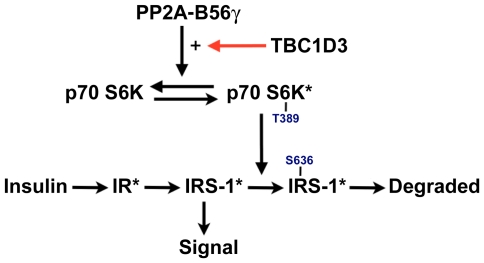
Model for the regulation of IRS-1 degradation by TBC1D3 expression. We propose that TBC1D3 suppresses the degradation of IRS-1 by regulating the phosphorylation of S6K at T389. In this model, mTOR phosphorylates S6K in response to insulin signaling. TBC1D3 interacts, directly or indirecly, with PP2A B56γ to enhance the dephosphorylation of S6K:T389 thereby reducing the S6K-dependent phosphorylation of IRS-1 at key sites which are required for IRS-1 ubiquitination and degradation.

Taken together, the reversal of TBC1D3-enhanced S6K phosphorylation by OA and by silencing PP2A B56γ supports the conclusion that PP2A B56γ is a prime downstream target of TBC1D3. Based on these early observations, we offer a working model (Figure 7) to guide further experimentation. In this model, insulin (or IGF1) activates IRS-1 and the Akt pathway to enhance nutrient accumulation. As proposed by Pan and colleagues [10], a negative feedback loop is created via the mTOR/S6K pathway to limit the response to insulin by enhancing the Cul7-dependent degradation of IRS-1. We propose that TBC1D3 sustains the response to insulin by delaying or suppressing the ubiquitination and degradation of IRS-1. This is accomplished by the combined action of TBC1D3 and PP2A B56γ that results in deactivating S6K by S6K:T389 dephosphorylation.

Notably, previous work showed that TBC1D3 also suppressed the recruitment of the E3 ligase CBL to the EGF receptor thereby suppressing the ubiquitination and degradation of the activated EGFR [6]. It is possible that by interacting with the PP2A B56γ complex, TBC1D3 may lead to selective de-phosphorylation of key elements involved in insulin, EGF and in other growth factor signaling pathways, leading to aberrant signaling and growth.

The human genome encodes a significant number of genes that are either human- or hominoid-specific and that have no known orthologs in lower organisms [50]. These genes and the proteins they encode are likely to reveal information about human evolution and possibly provide insight into many questions about human-specific physiology and pathophysiology. S6K and its upstream regulator mTOR and IRS-1 have been shown to play a role in aging and lifespan in model organisms [51]. By demonstrating that TBC1D3 modulates insulin signaling by altering S6K-dependent phosphorylation of IRS-1 via the B56γ subunit of PP2A, we forward the hypothesis that TBC1D3 plays a role, perhaps central, in human metabolism.

## Materials and Methods

### Reagents

Monoclonal α-tubulin, human insulin and MG132 were purchased from Sigma-Aldrich (St. Louis, MO). Antibodies against PKB/Akt (Akt) as well as phospho-Akt S473, phospho-MAPK, total MAPK, phospho-4EBP1, phospho-S636/639, phospho-S1101 and phospho-S307 of IRS-1, S6K and phospho-S6K were obtained from Cell Signaling Technology (Beverly, MA). Phospho-S270 of IRS-1 and PI3k-p85a antibody were obtained from Santa Cruz Biotechnology (Santa Cruz, CA). Antibody to phospho-S312 of IRS-1 was acquired from Biosource (Camarillo, CA). Monoclonal antibody against phospho-Tyr of IRS-1 was purchased from BD Transduction Laboratories (San Jose, CA). IRS-1 antibody was provided by Dr. Mike Mueckler, Washington University (St. Louis, MO). The monoclonal antibody directed against the C-terminal 50 amino acids of TBC1D3 was generated by the Hybridoma Center, Washington University (St. Louis, MO). A rabbit polyclonal antibody against PP2A B56γ3 was a gift from Dr. Bill Hahn, Dana Farber Cancer Institute (Boston, MA). pCLS2-IRS-1 construct was a gift from Dr. Jianping Ye, National Institutes of Health (Bethesda, MD). Rapamycin was purchased from Enzo Life Sciences (Plymouth Meeting, PA). pRK7-HA-S6K-ED_3_E was a gift from Dr. Tony Hunter, Salk Institute (La Jolla, CA) [48].

### PP2A B56γ and TBC1D3 silencing

PP2A B56γ was successfully suppressed using three different shRNAs against PP2A B56γ (Gene ID: NM_ 002719.X-385s1c1, NM_002719.X-1481s1c1 and NM_002719.X-1348s1c1) [14], provided by Genome Center at Washington University in St. Louis. Briefly, DU145 cells were plated at about 50% confluency and transfected with pLKO.1-puro shGFP or pLKO.1-puro vectors containing shRNAs targeting PP2A B56γ using Lipofectamine 2000. Suppression of PP2A B56γ was assessed after 72 hours of incubation by Western blotting [45]. TBC1D3 was successfully knocked down using two different siRNA against TBC1D3 (Ambion, Inc; Silencer Select Pre-designed siRNA ID# s230583 and ID# s230584). Briefly, cells were transfected with TBC1D3 siRNA (50 nM final concentration) or irrelevant siRNA (siGLO control siRNA, Dharmacon) using Lipofectamine RNAiMax and were used for the appropriate experiments after 36 hours. RNA extracted from cells using RNeasy (Qiagen, MA) was used to detect TBC1D3 transcript levels by quantitative real-time PCR. Briefly, first-strand cDNA was produced using SuperScript III Reverse Transcriptase (Life Technologies, CA) with random primers. 1 µg of total RNA was used for each reverse transcription reaction (20 µl). For the real-time PCR, 2 µl of the 20 µl reverse transcription reaction mixture were used. Forward primer 5′- GCATCGACCGGGACGTAAG- 3′; reverse primer 5′- CCTCCGGGTTGTACTCCTCAT -3′, corresponding to nucleotide 431 and 550, respectively), using Platinum Taq DNA Polymerase Hi-Fi (Life Technologies, CA). Real-time PCR was carried out using an ABI Prism® 7000 Sequence Detection System (Life Technologies, CA). Cycling conditions were as follows: denaturation (95°C for 5 min), amplification and quantification (95°C for 30 s, 55°C for 30 s, and 72°C for min), repeated 40 times. Each sample was run in triplicate.

### SDS-PAGE and Western blotting

To obtain cell lysates, cell monolayers were washed with PBS and lysed in ice-cold lysis buffer (PBS, 1% Triton X-100, 1 mM phenylmethylsulfonylfluoride, 2 µg/ml pepstatin A, 2 µg/ml leupeptin, and 2 µg/ml aprotinin). Cell lysates were clarified by centrifugation and proteins were resolved by SDS-PAGE and transferred to nitrocellulose membranes, which were blocked and probed with the indicated antibodies. To determine relative protein amounts, three representative exposures for each sample were quantified using AlphaEaseFc software (Alpha Innotech Corporation, San Leandro, CA).

### Signaling and degradation assays

Protein phosphorylation and IRS-1 degradation were measured in cells serum-starved for 5 hours. Cells were incubated in the presence of insulin (10 nM) at 37°C, washed with ice-cold PBS and lysed. Proteins were separated by SDS-PAGE and analyzed by Western blotting.

### Immunoprecipitation

Cells were lysed in ice-cold immunoprecipitation buffer containing 20 mM Tris/HCl, pH 7.5, 150 mM NaCl, 1% Triton X-100, 1 mM EGTA, 1 mM EDTA, 2.5 mM sodium pyrophosphate, 1 mM b-glycerolphosphate, 1 mM NaF, 1 mM sodium orthovanadate plus protease inhibitors. Extracts were immunoprecipitated by incubation with the appropriate antibody followed by immobilization on Protein G-Sepharose beads (Sigma). Beads were resuspended in sample buffer and analyzed by SDS-PAGE and Western blotting.

### PP2A activity assay

PP2A activity was assayed using PP2A Immunoprecipitation Phosphatase Assay Kit (Upstate). Briefly, PP2A catalytic (C) subunit was immunoprecipitated from 300 mg of cell lysates using anti-PP2A C subunit antibody (clone1D6, Upstate). Enzymatic activity was measured by incubation of the immunoprecipitated PP2A C subunit with the synthetic phosphopeptide K-R-pT-I-R-R following manufacturer's instructions.

### Statistical analysis

All experiments presented were repeated a minimum of three times. Results were expressed as mean values ± standard error (mean ± SE). Student's t-test or one-way analysis of variance (ANOVA) was performed to calculate statistical significance.
