# Correction: TBC1D3, a Hominoid-Specific Gene, Delays IRS-1 Degradation and Promotes Insulin Signaling by Modulating p70 S6 Kinase Activity

**DOI:** 10.1371/annotation/6ef317b6-c9eb-44a4-8e4d-556d064ce987

**Published:** 2012-03-29

**Authors:** Marisa J. Wainszelbaum, Jialu Liu, Chen Kong, Priya Srikanth, Dmitri Samovski, Xiong Su, Philip D. Stahl

There was an error in the first affiliation. The correct first affiliation is 1 Department of Cell Biology and Physiology, Washington University School of Medicine, St. Louis, Missouri, United States of America. 

